# Blood and Imaging Biomarkers in the Long-term Follow-up of Bicuspid Aortic Valve Patients

**DOI:** 10.1016/j.cjco.2023.09.012

**Published:** 2023-09-23

**Authors:** Zoë A. Keuning, Paul M. Hendriks, Anthonie L. Duijnhouwer, Frederike Meccanici, Hans-Marc J. Siebelink, Allard T. van den Hoven, Laurie W. Geenen, Jannet A. Eindhoven, Vivan J.M. Baggen, Judith A.A.E. Cuypers, Robert M. Kauling, Jolien W. Roos-Hesselink, Annemien E. van den Bosch

**Affiliations:** a Department of Cardiology, Erasmus MC, University Medical Centre Rotterdam, Rotterdam, The Netherlands; b Department of Cardiology, Radboud University Medical Centre, Nijmegen, The Netherlands; c Department of Cardiology, Leiden University Medical Centre, Leiden, The Netherlands; d ERN-GUARD-Heart: European Reference Network for Rare and Low Prevalence Complex Diseases of the Heart, Amsterdam, The Netherlands

## Abstract

**Background:**

Bicuspid aortic valve (BAV) is a common congenital heart defect. Patients with BAV are at risk for long-term complications such as valve stenosis and regurgitation. This study aimed to investigate sex differences in blood and imaging biomarkers and to describe the long-term prognostic value of blood and echocardiographic biomarkers.

**Methods:**

Patients were included from 2 prospective observational cohort studies; they underwent venous blood sampling and transthoracic echocardiography including speckle tracking. Analyzed blood biomarkers were red-cell distribution width (RDW), creatinine, C-reactive protein (CRP), troponin T, N-terminal pro B-type natriuretic peptide (NT-proBNP), and transforming growth factor-beta (TGF-β). Sex differences were analyzed at baseline. Associations between biomarkers and arrhythmia-free and intervention-free survival were determined by Cox regression, adjusted for age and sex.

**Results:**

A total of 182 patients with BAV were included: median age 34; interquartile range [IQR]: 23-46 years; 55.5% male. CRP, NT-proBNP, and RDW were higher in women, whereas creatinine, troponin T and TGF-β were higher among men. After a median follow-up time of 6.9 (IQR: 6.5-9.9) years, arrhythmia-free and intervention-free survival was, 81.0% and 73.1%, respectively. NT-proBNP was associated with both arrhythmia-free and intervention-free survival (hazard ratio [HR], 1.94, *P* = 0.005 and HR, 2.06, *P* = 0.002, respectively). On echocardiography higher left atrial (LA) size, left ventricular end-diastolic diameter (LVEDD), left ventricular (LV) mass index and E/e’ ratio were associated with lower arrhythmia-free survival, whereas higher LA size, LV mass index, aortic valve peak velocity, and aortic regurgitation were associated with lower intervention-free survival.

**Conclusions:**

Differences were observed in blood biomarkers between men and women with BAV. Besides LV systolic parameters, diastolic LV function and NT-proBNP should have a more prominent role as prognostic markers in clinical care.

## Résumé

**Contexte:**

La bicuspide valvulaire aortique (BVA) est une anomalie cardiaque congénitale fréquente. Les patients atteints d’une BVA présentent des risques de complications à long terme, comme la sténose valvulaire ou la régurgitation valvulaire. Cette étude visait 1) à évaluer les différences entre les sexes en ce qui concerne les biomarqueurs sanguins et les biomarqueurs à l’imagerie; et 2) à décrire la valeur pronostique à long terme des biomarqueurs sanguins et échocardiographiques.

**Méthodologie:**

Des patients de 2 études de cohortes observationnelles prospectives ont été inclus dans l’analyse. Des échantillons de sang veineux ont été prélevés, et des échocardiographies transthoraciques, y compris le suivi des marqueurs acoustiques, ont été effectuées. Les biomarqueurs sanguins analysés étaient les suivants : indice de distribution des globules rouges (IDR), créatinine, protéine C-réactive (CRP), troponine T, propeptide natriurétique de type B N-terminal (NT-proBNP) et facteur de croissance transformant β (TGF-β). Les différences entre les sexes ont été analysées au départ. Les liens entre les biomarqueurs et la survie sans arythmie et sans intervention ont été déterminés par la régression de Cox, avec correction en fonction de l’âge et du sexe.

**Résultats:**

Cent quatre-vingt-deux patients présentant une BVA étaient inclus (âge médian de 34 [écart interquartile : 23-46] ans, 55,5 % hommes). La CRP, la NT-proBNP et l’IDR étaient plus élevées chez les femmes, alors que la créatinine, la troponine T et le TGF-β étaient plus élevés chez les hommes. Après une période de suivi médiane de 6,9 (écart interquartile : 6,5-9,9) ans, les taux de survie sans arythmie et sans intervention étaient respectivement de 81,0 % et de 73,1 %. La NT-proBNP a été associée à la survie sans arythmie (rapport des risques instantanés [RRI] : 1,94, *p* = 0,005) et à la survie sans intervention (RRI : 2,06, *p* = 0,002). À l’échocardiographie, des valeurs élevées pour la taille de l’oreillette gauche, le diamètre télédiastolique du ventricule gauche (VG), l’indice de masse du VG et le rapport E/e’ étaient associées à un faible taux de survie sans arythmie, alors que des valeurs élevées pour la taille de l’oreillette gauche, l’indice de masse du VG, la vitesse maximale aortique et la régurgitation aortique étaient associées à un faible taux de survie sans intervention.

**Conclusions:**

Les biomarqueurs sanguins variaient en fonction du sexe des personnes présentant une BVA. Outre les paramètres systoliques du VG, la fonction VG diastolique et la NT-proBNP devraient être davantage utilisées comme marqueurs pronostiques en soins cliniques.

Bicuspid aortic valve (BAV) is a common congenital cardiac defect with a prevalence of 0.5% to 2.0% in the general population.^1^, ^2^, ^3^ Although the mortality rate in young patients with BAV is low, aortic valve replacement (AVR) is often necessary at a young age caused by the development of severe aortic valve (AV) dysfunction. Moreover, patients with BAV are at high risk to develop complications such as arrhythmias and heart failure, which leads to an important health care burden.^4^^,^^5^

It has long been acknowledged that BAV is more prevalent in men than in women in a ratio of 3:1.^6^ Previous studies investigating male-female differences in patients with BAV described differences in type of AV dysfunction and morbidity rates between men and women, with men experiencing more cardiac events.^7^^,^^8^ Although these studies underlined the importance of research into sex differences in patients with BAV, data describing differences in prognostic markers between male and female patients with BAV are limited.

As the clinical presentation in patients with BAV varies greatly, early identification of high-risk patients is challenging, and the search for reliable prognostic factors is ongoing. Literature on the prognostic relevance of circulating blood biomarkers in patients with BAV is scarce and mostly focused on aortopathy instead of left ventricular (LV) remodelling.^9^ Previous research by our group demonstrated that N terminal pro B-type natriuretic peptide (NT-proBNP), C-reactive protein (CRP), and troponin T levels are elevated in patients with BAV, whereas transforming growth factor-β1 (TFG- β1) was significantly lower compared with controls.^10^ However, the prognostic relevance of these blood biomarkers, alongside the traditional echocardiographic parameters, remains to be investigated.

Therefore, this prospective study aimed to evaluate male-female differences for these blood biomarkers and investigate the prognostic value of blood biomarkers for long-term outcome in patients with BAV in relation to traditional echocardiographic parameters.

## Methods

### Study design and population

In this study, data on patients with BAV were extracted from 2 prospective studies: the BIOmarkers in CONgenital heart disease (BioCon) study, a single-centre prospective observational cohort study that included patients from the outpatient clinic with moderate or complex congenital heart disease (CHD) between 2011 and 2013 and the BAV-cohort, a multicentre observational cohort study that included patients with BAV or Turner syndrome between 2014 and 2016. The study protocols have been described in more detail previously.^11^^,^^12^ In the current study, patients with BAV were selected from the 2 cohorts and combined in a new database. Patients with previous AVRs were excluded. Both studies were approved by the Medical Ethics Committee (MEC10-165 and MEC14-225), and written informed consent was obtained from all participants. The study was performed according to the declaration of Helsinki.

### Study endpoints

All patients were evaluated for the occurrence of the following events: arrhythmias, heart failure (requiring hospitalization or change in medication), surgical or percutaneous aortic valve intervention, and death. Supraventricular arrhythmias were included when symptomatic and documented; ventricular arrhythmias were included when documented, with or without symptoms. Postoperative arrhythmias that occurred during the hospitalization for intervention were not included as arrhythmias. Atrial and ventricular premature complexes were not considered arrhythmias. The primary endpoints were arrhythmia- and intervention-free survival. Heart failure-free survival was not analyzed separately in Cox regression because of the low number of events. Event-free survival was defined as freedom of any of the aforementioned events. Assessment of the endpoints was performed blinded to blood biomarker levels and echocardiography. Follow-up of survival was checked using the Municipal Population Register and was 100% complete. Patients who did not reach the endpoints were right-censored on April 1, 2022.

### Laboratory testing

We selected the following biomarkers based on the available measurements in both cohorts: red cell distribution width (RDW), creatinine, NT-proBNP, CRP, troponin T and TGF-β1. Venous blood samples were obtained after a minimum of 30 minutes of rest. Blood samples were solely used for study purposes; clinical decision making was irrespective of blood biomarker levels. RDW was measured in fresh K2EDTA plasma samples using Sy smex XN-1000 Hematology Analyzer (Sysmex Europe GmbH, Norderstedt, Germany). NT-proBNP and creatinine were measured in fresh blood by a commercial electrochemiluminescende immunoassay (Roche Diagnostics, Rotkreuz, Switzerland) and a commercial colorimetric quantitative assay (Roche Diagnostics, Rotzkreuz, Switzerland), respectively. Blood samples for other measurements were aliquoted and stored at –80 °C within 2 hours after withdrawal. Serum high-sensitivity (hs)-CRP and hs-troponin T were measured in thawed blood samples using respectively Roche immunoturbidimetric assays and electrochemiluminescence immunoassays (Roche Diagnostics, Basel, Switzerland). TGF-β1 measurements were only performed in the BAV cohort. Serum concentration was measured using quantitative sandwich enzyme linked immunosorbent assay (ELISA) as instructed by the manufacturer (Duoset, ELISA, R&D Systems Europe, Ltd, Abingdon, United Kingdom). Beforehand, acid activation and neutralization was used to active the latent TGF-β1 in the patients’ sera.

### Echocardiography

Standard 2-dimensional transthoracic echocardiogram (2D TTE) in harmonic imaging was acquired from all patients by an experienced sonographer using an iE33 or EPIQ7 ultrasound system (Philips Medical Systems, Best, The Netherlands) with a broadband X5-1 matrix-array transducer (composed of 3040 elements with 1-5MHz extended operating frequency range). Aortic stenosis was defined as aortic peak velocity ≥ 2.5 m/sec. Conventional measurements of the LV and degree of aortic stenosis and regurgitation were classified according to the recommendations of the American Society of Echocardiography and the European Association of Cardiovascular Imaging.^13^ LA volume, left ventricular end-diastolic diameter (LVEDD), and left ventricular end-systolic diameter (LVESD) were indexed according to body surface area. Speckle-tracking analysis was performed using dedicated commercially available software (2D Cardiac Performance Analysis, Tomtec Imaging Systems, Unterschleissheim, Germany). Global longitudinal strain was measured using 2D images in 2-, 3-, and 4-chamber views, as available.

### Statistical analysis

Continuous variables were presented as mean ± standard deviation or median (interquartile range [IQR]) according to their distribution. Categorical variables were presented as cases (percentage). Continuous variables were compared using an unpaired Student's *t*-test for normally distributed data or a Mann-Whitney U test for non-normally distributed data. Survival curves were based on the Kaplan-Meier estimator and compared using the log-rank test. Blood biomarkers were log2 transformed to obtain normal distribution. Blood biomarkers and echocardiographic parameters were standardized into Z-scores to be comparable in effect size. Missing values were imputed for survival analysis using multiple imputation via chained equations with 10 imputed datasets and 25 iterations. Associations with arrhythmia-free survival and intervention-free survival were evaluated using multivariable Cox-proportional hazard modelling adjusted for age and sex. A difference in the association between blood and imaging biomarkers and the endpoint per sex was evaluated using interaction terms. The likelihood ratio test was used to compare models. A sensitivity analysis excluding patients with Turner syndrome and a history of aortic coarctation was performed. A 2-sided *P* value < 0.05 was considered statistically significant. The Bonferroni-Holm method was used to account for multiple testing. SPSS (SPSS Statistics for Windows, Version 28.0. and R version 4.0.5, IBM Corp [Released 2017] Armonk, New York, USA) packages "survival," "mice," "ggplot2," and "metafor" were used for statistical analyses.

## Results

**Figure 1:**
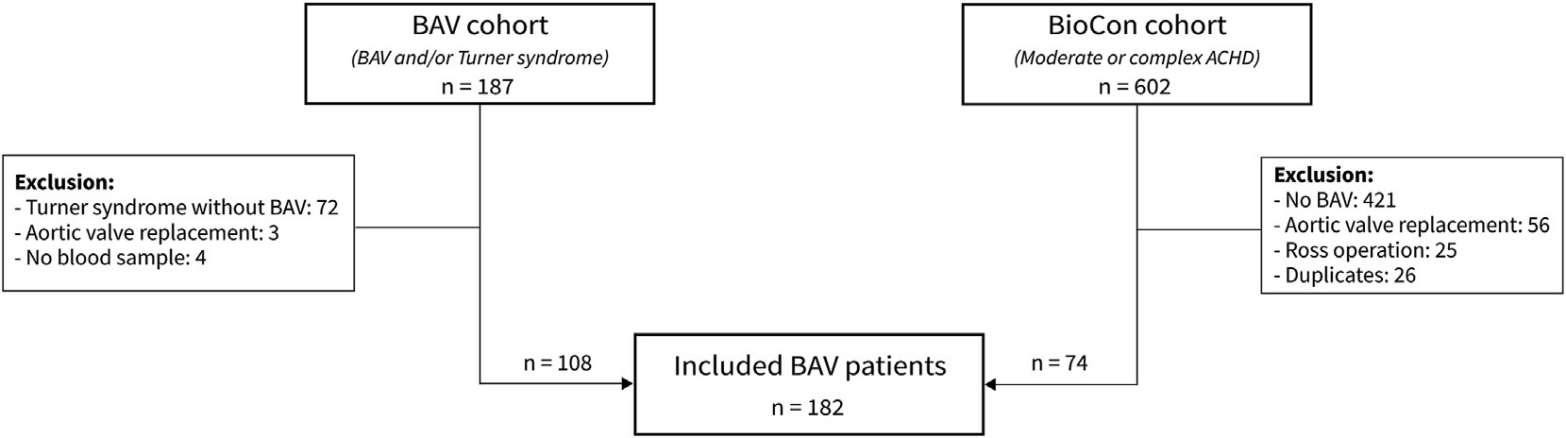
Flowchart of inclusion from BioCon and BAV cohort. ACHD, adult congenital heart disease; BAV, bicuspid aortic valve.

A total of 182 patients with BAV were included from the BioCon and BAV cohorts (Fig. 1). The median age was 34 (IQR: 23-46) years, and 55.5% of patients were male (Table 1). Demographic parameters, blood, and imaging biomarkers at baseline are described in Table 1. Most patients had Sievers type 1 BAV (78.6%), and 59 (32.4%) had histories of aortic coarctation. Twenty-seven (14.8%) patients had previous balloon valvuloplasties or surgical valvulotomies. The left atrium (LA) was enlarged in 23% of patients. Moderate or severe aortic valve stenosis was present in 64 (35.2%) patients and regurgitation in 79 (43.4%) patients.

**Table 1:** Baseline characteristics

	Valid cases n (%)	All patients n = 182	Male n = 101	Female n = 81	*P* value
**Demographic characteristics**					
Age, years	182 (100)	34 (23-46)	31 (22-46)	35 (23-45.5)	0.446
BSA, m^2^	182 (100)	1.9 ± 0.3	2.0 ± 0.2	1.7 ± 0.2	< 0.001
Systolic blood pressure, mm Hg	180 (98.9)	126 (115-136)	128 (118-136)	125 (114-136)	0.151
Heart rate, bpm	177 (97.2)	72 ± 14	70 ± 14	74 ± 14	0.140
Turner syndrome, n (%)	182 (100)	22 (12.1)	0 (0)	22 (27.2)	
Sievers classification, n (%)	182 (100)				0.719
Type 0		27 (14.8)	15 (14.9)	12 (14.8)	
Type 1		143 (78.6)	78 (77.2)	65 (80.2)	
Type 2		12 (6.6)	8 (7.9)	4 (4.9)	
History of coarctation, n (%)	182 (100)	59 (32.4)	31 (30.7)	28 (34.6)	0.579
Prior AV intervention∗, n (%)	182 (100)	27 (14.8)	17 (16.8)	10 (12.3)	0.587
**Blood biomarkers**					
RDW, %	149 (81.9)	12.7 (12.3-13.1)	12.6 (12.3-13.0)	12.8 (12.4-13.3)	0.041
Creatinine, μmol/L	165 (90.7)	76 ± 14.5	83 ± 11	68 ± 14	< 0.001
CRP, mg/L	182 (100)	1.3 (0.5-2.7)	0.9 (0.4-1.7)	1.7 (0.8-3.8)	< 0.001
Troponin T, ng/L	182 (100)	4.1 (1.5-7.0)	5.0 (3.0-8.0)	3.0 (1.5-6.0)	< 0.001
NT-proBNP, pmol/L	161 (88.5)	6.7 (3.0-12.9)	4.2 (2.3-8.3)	11.0 (5.2-18.0)	< 0.001
TGF-β, ng/L	108 (59.3)	9.7 (8.2-11.4)	10.0 (8.6-12.2)	9.3 (7.4-10.5)	0.025
**Echocardiography**					
LA volume index > 34 mL/m^2^	161 (88.5)	37 (23.0)	25 (28.7)	12 (16.2)	0.060
LVEDD indexed, mm/m^2^	178 (97.8)	26.8 ± 3.4	27.9 ± 3.4	25.9 ± 3.1	0.310
LVESD indexed, mm/m^2^	170 (93.4)	16.9 ± 3.3	17.7 ± 3.1	16.3 ± 3.5	0.671
E/A ratio	164 (90.1)	1.4 (1.0-1.8)	1.4 (1.0-2.0)	1.3 (1.0-1.6)	0.182
E/e’ ratio	160 (87.9)	9.0 (6.8-12.7)	8.1 (6.3-11.6)	10.1 (7.6-13.6)	0.005
LV ejection fraction, %	157 (86.3)	58.9 ± 6.5	57.9 ± 6.8	60.0 ± 5.9	0.045
LV GLS, %	128 (70.3)	–17.2 ± 2.9	–16.7 ± 2.9	-17.9 ± 2.8	0.019
LV mass index, g/m^2^	172 (94.5)	88 (73-107)	97 (80-113)	77 (66-97)	< 0.001
LV hypertrophy, n (%)	172 (94.5)	40 (23.3)	20 (20.8)	20 (26.3)	0.398
Aortic valve peak velocity, m/s	182 (100)	2.2 (1.6-3.3)	2.2 (1.6-2.2)	2.3 (1.6-3.2)	0.793
Aortic valve regurgitation, n (%)	182 (100)				0.068
No		62 (34.1)	26 (25.7)	36 (44.4)	
Mild		70 (38.5)	43 (42.6)	27 (33.3)	
Moderate		41 (22.5)	26 (25.7)	15 (18.5)	
Severe		9 (4.9)	6 (5.9)	3 (3.7)	
Aortic valve stenosis, n (%)	182 (100)				0.935
No		103 (56.6)	56 (55.4)	47 (58.0)	
Mild		15 (8.2)	8 (7.9)	7 (8.6)	
Moderate		40 (22.0)	24 (23.8)	16 (19.8)	
Severe		24 (13.2)	13 (12.9)	11 (13.6)	
Ascending aorta diameter, mm	178 (97.8)	36.5 ± 7.6	37.9 ± 7.8	34.7 ± 7.0	0.005

AV, aortic valve; bpm, beats per minute; BSA, body surface area; CRP, C-reactive protein; GLS, global longitudinal strain; LA, left atrium; LV, left ventricle; LVEDD, left ventricular end-diastolic diameter; LVESD, left ventricular end-systolic diameter; RDW, red-cell distribution width; TGF-beta, transforming growth factor-beta1.

∗ Previous AV intervention includes surgical valve repair and percutaneous balloon valvuloplasty.

### Sex differences in BAV

**Figure 2:**
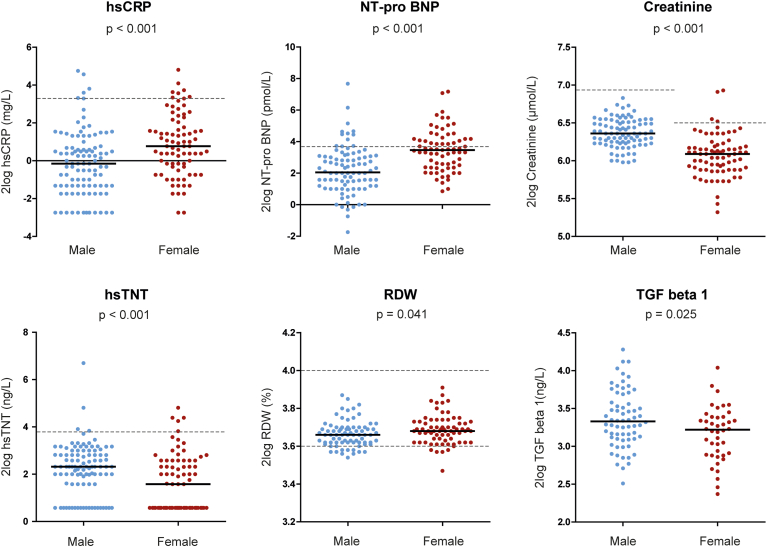
Biomarker levels in male and female patients with bicuspid aortic valve (BAV). Biomarker levels are presented at the *y*-axis in the 2log scale. The **thick black line** represents the median value. The **dashed line** represents the upper limit of normal (hsCRP 10 mg/L, NT-proBNP 14 pmol/L, hsTNT 14 ng/L, creatinine for men 115 μmol/L and for women 95 μmol/L) and the lower and upper limit of RDW (12.0%-16.0%). hsCRP, high-sensitive C-reactive protein; hsTNT, high-sensitive troponin T; NT-pro BNP, N-terminal pro B-type natriuretic peptide; RDW, red-cell distribution width; TGF-β1, transforming growth factor β1.

A significant difference was found for all blood biomarkers between men and women (Table 1). Blood biomarker levels for both men and women are depicted in Figure 2. RDW was lower in men than in women (12.6% vs 12.8%, *P* = 0.041), whereas creatinine levels were significantly higher among men (83 μmol/L vs 68 μmol/L, *P* < 0.001). CRP levels were lower in men (0.9 mg/L vs 1.7 mg/L, *P* < 0.001), and troponin T levels were significantly higher than in women (5.0 ng/L vs 3.0 ng/L, *P* < 0.001). NT-proBNP levels were lower in men (4.2 pmol/L vs 11.0 pmol/L, *P* < 0.001). In the overall population, NT-proBNP was elevated (defined as > 14 pmol/L) in 22% of the patients and was more often elevated in women than men (33.3% vs 8.9%, *P* < 0.001).

Indexed LV dimensions were similar in men and women. LV function was slightly worse in men compared with women, with a LVEF of, respectively, 57.9% ± 6.8% vs 60.0% ± 5.9% and global longitudinal strain (GLS) of –16.7% ± 2.9% vs –17.9% ± 2.8%, respectively.

### Survival

**Figure 3:**
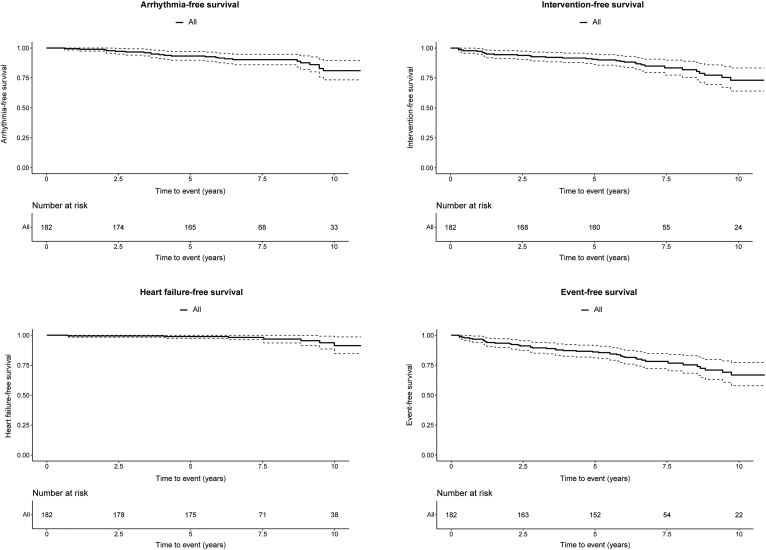
Kaplan-Meier curves of arrhythmia-free survival, intervention-free survival, event-free survival, and heart failure-free survival.

**Figure 4:**
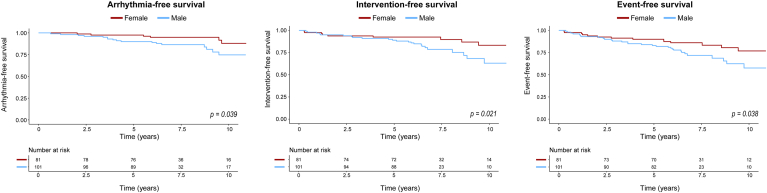
Arrhythmia-free, intervention-free, and event-free survival compared between male and female patients.

During a median follow-up duration of 6.9 (IQR: 6.5-9.9) years, 44 patients experienced events. Three (1.6%) patients died, 5 (2.7%) developed heart failure, 21 (11.5%) experienced arrhythmias, and 31 (17.0%) underwent AV interventions. A detailed overview of all events can be found in Supplemental Table S1. During 10-year clinical follow-up, rates for heart failure-free, arrhythmia-free, and intervention-free survival of the overall population were 91.3%, 81.0%, and 73.1%, respectively (Fig. 3). Event-free survival for the overall population at 10-year follow-up was 66.8%. Sex-stratified survival curves are depicted in Figure 4. We observed more arrhythmias in men than in women, with arrhythmia-free survival rates of 74.7% compared with 83.3% (*P* = 0.039). Intervention-free survival was also significantly lower in men than in women (63.1 vs 83.3%, *P* = 0.021).

### Prognostic factors

**Figure 5:**
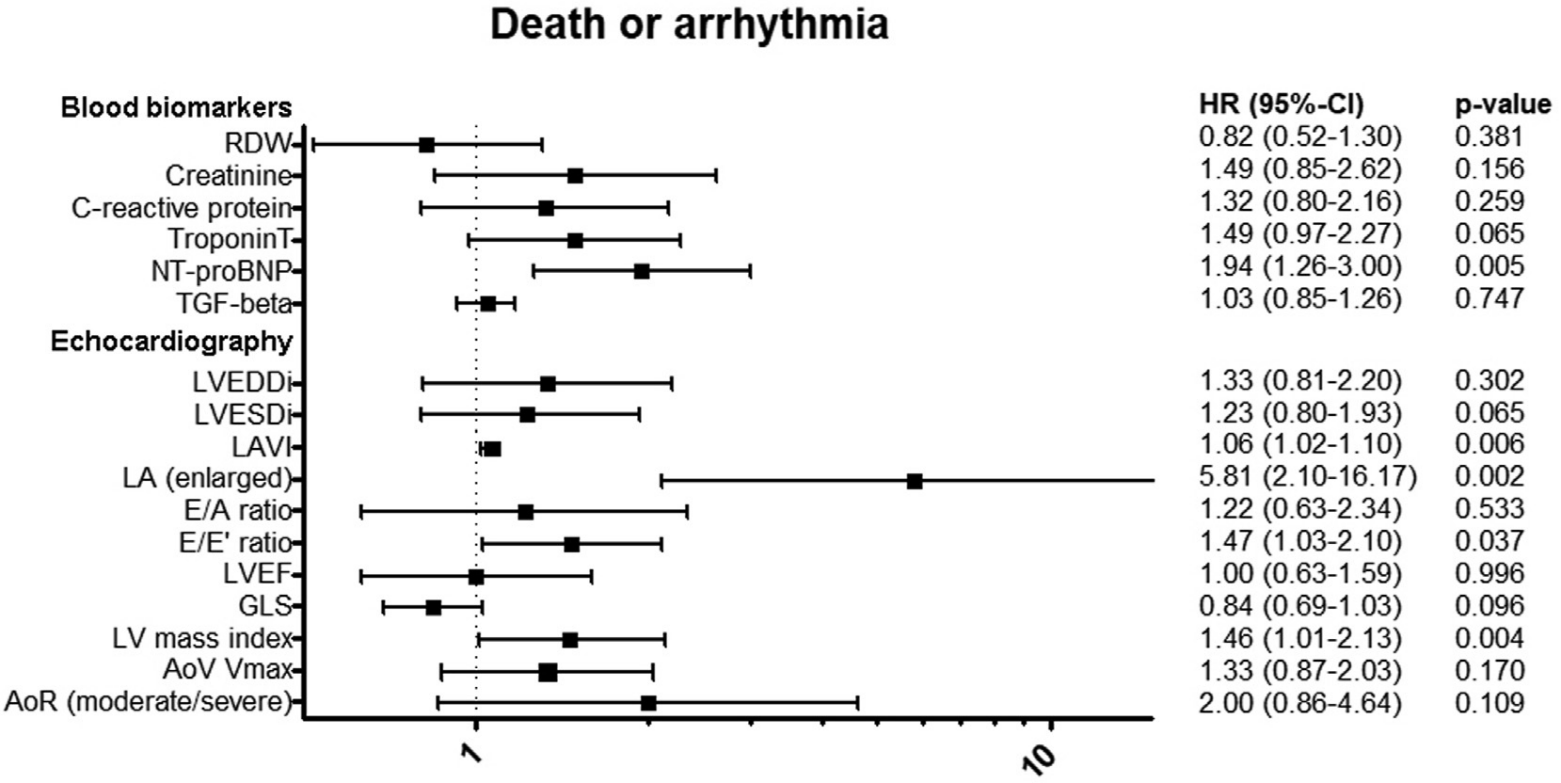
Association among composite endpoint of death or arrhythmia, blood biomarkers, and echocardiographic characteristics. HRs are standardized and represent HR per standard deviation increase, with exception of categorical covariates (LA [enlarged], aortic regurgitation). Blood biomarkers are 2log transformed. All HRs are obtained from multivariable Cox-proportional hazards (ph) models and adjusted for age and sex. AoR, aortic valve regurgitation; AoV, aortic valve; GLS, global longitudinal strain; HRs, hazard ratios; LA, left atrium; LAVi, left atrial volume indexed; LVEDDi, left ventricular end diastolic diameter indexed; LVEF, left ventricular ejection fraction; LVESDi, left ventricular end systolic diameter indexed; RDW, red-cell distribution width; TGF-β1, transforming growth factor beta1, V_max_, maximum velocity.

**Figure 6:**
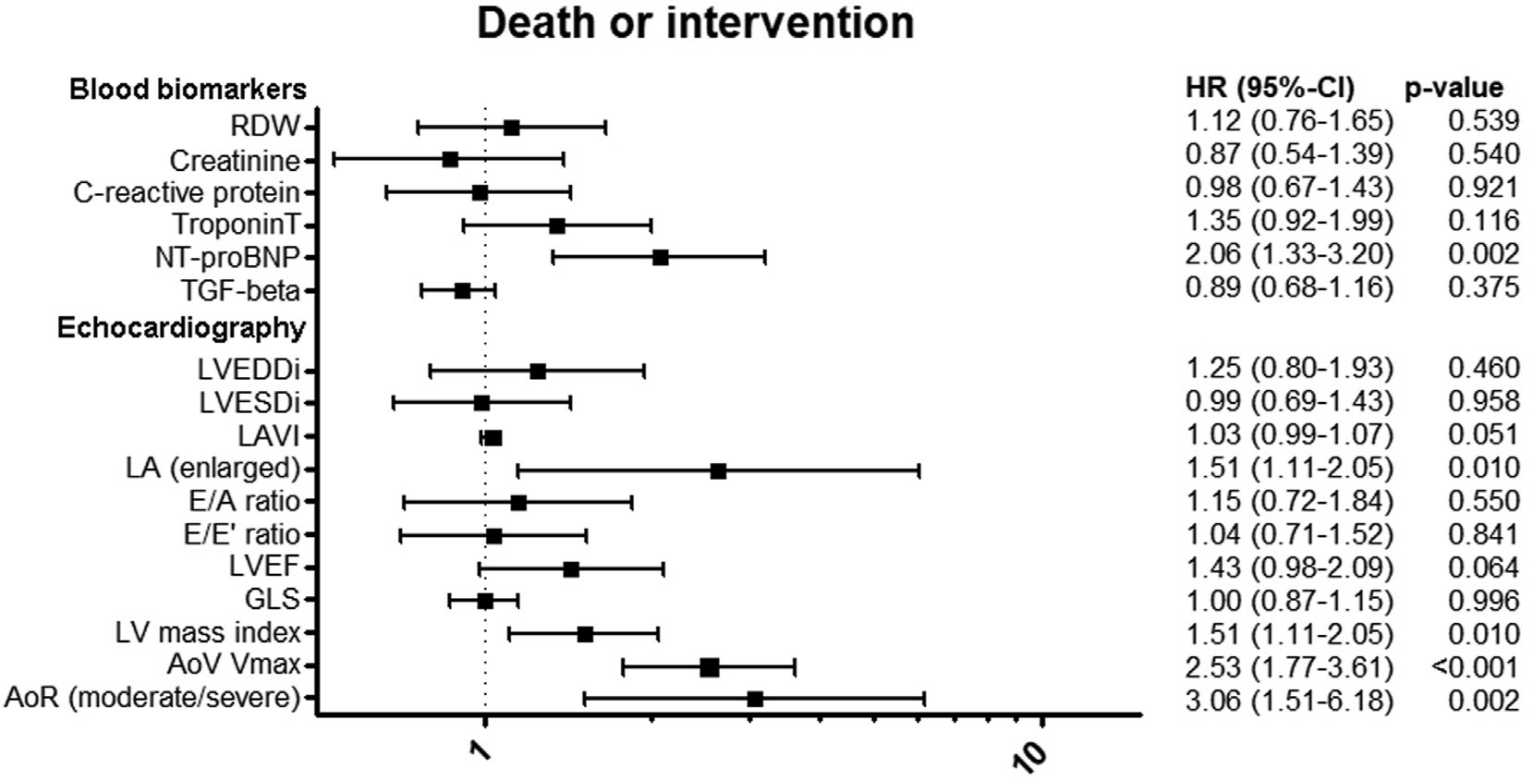
Association among composite endpoint of death or intervention, blood biomarkers, and echocardiographic characteristics. HRs are standardized and represent HR per standard deviation increase, with exception of categorical covariates (LA [enlarged], aortic regurgitation). Blood biomarkers are 2log transformed. All HRs are obtained from multivariable Cox-ph models and adjusted for age and sex. AoR, aortic valve regurgitation; AoV, aortic valve; GLS, global longitudinal strain; HR, hazard ratio; LA, left atrium; LAVI, left atrial volume indexed; LVEDDi, left ventricular end-diastolic diameter indexed; LVEF, left ventricular ejection fraction; LVESDi, left ventricular end-systolic diameter indexed, RDW, red-cell distribution width; TGF-β1, transforming growth factor beta1, V_max_, maximum velocity.

Figures 5 and 6 show the associations between clinical factors and arrhythmia-free and intervention-free survival, adjusted for age and sex, respectively. Of the blood biomarkers, higher NT-proBNP levels showed the strongest association with both the composite endpoint of death or arrhythmia (hazard ratio [HR], 1.94; 95% confidence interval [CI], 1.26-3.00) and death or intervention (HR, 2.06, 95% CI, 1.33-3.20). RDW, creatinine, CRP, and TGF-β1 were not associated with the endpoints. There were no differences in the magnitude of the prognostic value of blood biomarkers and endpoints between men and women (Supplemental Table S2).

Arrhythmia-free survival was associated with increased indexed LA volume (HR, 1.06; 95% CI, 1.02-1.10) and E/e’ ratio (HR, 1.47; 95% CI, 1.03-2.10). However, when adjusted for multiple testing, no significant association was found between E/e’ ratio and death or arrhythmia (Supplemental Tables S3 and S4). An increase in LV mass index was associated with lower arrhythmia-free survival (HR, 1.46; 95% CI, 1.01-2.13) and intervention-free survival (HR, 1.51; 95% CI, 1.11-2.05). Moreover, increased LA volume, higher aortic valve peak velocity, and the presence of aortic regurgitation were associated with lower intervention-free survival. No association was found between left ventricular ejection fraction (LVEF) and LV GLS and arrhythmia-free survival or intervention-free survival. Sensitivity analysis excluding patients with Turner syndrome and patients with a history of aortic coarctation found no differences in results besides a significant association between GLS and arrhythmia-free survival and GLS and LVEF and intervention-free survival (Supplemental Table S5).

## Discussion

This prospective study evaluated the prognostic value of blood biomarkers in patients with BAV over a median follow-up period of 6.9 years. Marked differences were found in blood biomarker levels between men and women at baseline. Moreover, sex differences were found for long-term outcomes, as male patients with BAV had significantly lower event-free survival than female patients. The most prominent prognostic blood biomarker for both arrhythmia-free survival and intervention-free survival was NT-proBNP, whereas the most important prognostic echocardiographic biomarkers were LV mass index and indicators for diastolic LV dysfunction, especially LA enlargement.

### Blood biomarkers

Our study demonstrated significant sex differences in blood biomarkers in patients with BAV. Previous studies already described sex differences in blood biomarkers in the general population. There are several physiological mechanisms that could cause these differences: for example, differences in sex hormones and sex hormone receptor expression.^14^^,^^15^ Differences in androgen levels are most pronounced between premenopausal women and men of comparable age. Especially in our young population, it can therefore be expected that the hormonal effects are prominent.^15^ Furthermore, the introduction of high-sensitivity assays may have increased the possibility to detect—in particular, more subtle—sex differences.^16^^,^^17^ The variety in circulating blood biomarker levels between male and female patients that could be caused by underlying physiological mechanisms gives rise to the question whether sex-specific cutoff values should be implemented to optimize patient-specific care.^17^

NT-proBNP was elevated in 22% of all patients, and we observed higher NT-proBNP levels in women than in men. This finding is in line with previous literature showing higher NT-proBNP levels in healthy women.^18^^,^^19^ Testosterone could upregulate neprilysine activity, thereby decreasing circulating NT-proBNP levels.^20^ Estrogens, on the other hand, have shown to increase natriuretic peptide gene expression.^20^ Marked differences in troponin T levels using high-sensitive assays have been observed between men and women, with higher levels found in men. These findings are consistent with current literature.^16^^,^^17^ Increased cardiac mass in men could attribute to this as well as underlying hormonal differences that play a role in cardiomyocyte apoptosis.^20^ CRP levels represent inflammation. It is known that ovulation is accompanied by low-grade inflammation and increased CRP levels have been observed during ovulation.^21^^,^^22^ We believe that the significant sex-related differences in blood biomarker levels found in our study are similar to findings in a healthy population and that these are not specific to BAV. However, except for creatinine and RDW, these differences are not taken into account in clinical practice. Ignoring these sex-specific findings could lead to overtreatment or undertreatment.

### Survival

We observed an event-free survival of 66.8% after a median follow-up of 6.9 years. Taking into consideration that the overall mortality was low, this event-free survival mainly represents the need for intervention and the occurrence of arrhythmias in our BAV population. AV (re)intervention was necessary in 17% of patients. This is similar to previous studies that included patients with BAV of similar age and reported isolated AV intervention in 19% to 27% of patients during a follow-up of 8 to 13 years.^4^^,^^5^^,^^23^ Moreover, we found a relatively high rate of arrhythmias, with 1 in 10 patients experiencing arrhythmias (mainly of atrial origin), independent of the severity of stenosis. This is higher than reported in previous studies that described a prevalence of arrhythmias of 5% in patients with BAV of similar age.^4^^,^^5^ This difference may be explained by the variety in definitions for arrhythmia events or the complex patient group that was included in our study, as history of aortic coarctation and previous AV intervention were relatively common in our study population. Nonetheless, it does show that the arrhythmic burden in patients with BAV should not be underestimated. Our study population is young (median age 34 years), and the arrhythmogenic burden is only expected to increase over time.

Event-free survival was significantly lower in men compared with women. Previous literature also reported higher morbidity in male patients with BAV and higher regurgitation rates and more complications of the ascending aorta.^8^^,^^24^ In our study, we observed the same trend regarding aortic regurgitation. However, the higher burden of arrhythmias has not been described previously. A possible mechanism for this could be that higher regurgitation rates result in larger LA volume, eventually leading to atrial arrhythmias.

### Prognostic factors

Although the additive value of NT-proBNP in patients with atrial stenosis (AS) has been described previously,^25^ data in patients with BAV are lacking. In this study, NT-proBNP showed to be a prognostic factor for both arrhythmia-free and intervention-free survival. Higher levels of NT-proBNP probably represent higher myocardial workload in an early stage of disease, which can lead to arrhythmias and can identify the need for intervention over time. Despite sex-related differences in blood biomarker levels, we did not find a difference in the strength of the prognostic power of a difference in those biomarkers for any of our endpoints between men and women.

Prognostic echocardiographic parameters that have been studied are mostly focused on the aortic valve and systolic LV function. However, for complications such as arrhythmias it is debatable whether this is the way to go. A recent large registry study showed the prognostic importance of LA dilatation in patients with BAV and moderate or severe aortic regurgitation.^26^ In our study, LA enlargement also showed a significant prognostic association with arrhythmia-free survival, as did E/e’ ratio and LV mass index. GLS and LVEF were not associated with arrhythmia-free survival. Only in our sensitivity analysis, parameters of systolic function were associated with our endpoints. These findings, together with our results that NT-pro BNP is a prognostic biomarker, could indicate that diastolic LV function and LV remodelling are as important to monitor as AV peak velocity, as has been suggested before in degenerative valve diseases.^27^ LV systolic function is often still preserved in patients, whereas diastolic dysfunction and LV remodelling could be early markers for progression of disease.^28^^,^^29^

### Limitations

There are several limitations to this study. This study is based on 2 prospective cohorts with different inclusion criteria. Moreover, it was performed in a tertiary centre, and the patient population was relatively complex. There was no control cohort available with which to compare the observed sex-related differences, but these are similar to observations found in the literature of both diseased and healthy populations. Although the general BAV population is a heterogeneous population, and concomitant lesions are common, the percentages of comorbidities as aortic coarctation and previous interventions were relatively high in our study cohort. Therefore, this should be taken into account when generalizing our study results to an uncomplicated BAV population.

### Clinical implications

The BAV population shows great variety in course of disease. As a result, reliable identification of high-risk patients and determining the optimal timing for valve intervention is challenging. Current guidelines describe the development of symptoms and a decrease in LVEF as indicators for AVR.^30^ In our study however, echocardiographic parameters for LV diastolic dysfunction and LV remodelling were also of prognostic value, as was NT-proBNP. NT-proBNP can be an important prognostic marker that should be included in clinical care. However, as we observed profound sex differences in blood biomarkers at baseline, sex-specific thresholds for NT-proBNP should be considered. Finally, this study does indicate that in further research in patients with BAV, attention must be given to male-female differences.

## Conclusions

Although the mortality in patients with BAV is low, (re)interventions are often necessary, and arrhythmias are a common complication. Between men and women, profound differences in blood biomarker levels were observed at baseline. NT-proBNP and echocardiographic markers for LV diastolic dysfunction and LV remodelling are of prognostic value in patients with BAV for arrhythmia- and intervention-free survival and should have a more prominent role in clinical practice.
