# Supplementary material for: Blood and Imaging Biomarkers in the Long-term Follow-up of Bicuspid Aortic Valve Patients
Source: CJC Open. 2023 Sep 23;6(1):1–10. doi: 10.1016/j.cjco.2023.09.012 (PMC10837668; doi:10.1016/j.cjco.2023.09.012)
Supplement: Supplemental material [file mmc1.pdf]

SUPPLEMENTARY MATERIAL

Supplemental Table S1: Overview of events

**Event**	**N (%)**
Any event	44 (24.2)
Death<br>Cardiogenic shock<br>Unknown	3 (1.6)<br>1 (33.3)<br>2 (66.7)
Heart failure	5 (2.7)
Arrhythmia<br>Atrial fibrillation<br>SVT, unspecified<br>AV-nodal block<br>AV(N)RT<br>Ventricular extrasystole (>10%)<br>Ventricular tachycardia<br>Ventricular fibrillation	21 (11.5)<br>12 (57.1)<br>2 (9.5)<br>1 (4.8)<br>1 (4.8)<br>1 (4.8)<br>3 (14.3)<br>1 (4.8)
Intervention<br>Isolated aortic valve replacement<br>Bentall procedure<br>Primary aortic indication<br>Percutaneous balloon dilatation<br>Transcatheter aortic valve implantation	31 (17.0)<br>17 (54.8)<br>12 (38.8)<br>7 (58.3)<br>1 (3.2)<br>1 (3.2)
**Number of endpoints reached**	
One endpoint	30 (68.2)
Two endpoints	12 (27.2)
Three endpoints	1 (2.3)
Four endpoints	1 (2.3)

Supplemental Table S2: Beta-estimates of interaction terms between sex and blood biomarkers.

	**Death or arrhythmia**		**Death or intervention**	
**Interaction term with sex**	**Beta-estimate (95%-CI)**	**p-value**	**Beta-estimate (95%-CI)**	**p-value**
RDW	0.63 (-0.46-1.72)	0.238	-0.13 (-0.93-0.66)	0.731
Creatinine	0.29 (-0.85-1.43)	0.600	-0.48 (-1.43-0.46)	0.301
CRP	-0.86 (-1.93-0.22)	0.136	-0.67 (-1.50-0.16)	0.124
Troponin T	-0.23 (-1.15-0.69)	0.610	-0.22 (-0.96-0.52)	0.548
NT-proBNP	-0.06 (-1.08-0.97)	0.909	0.09 (-0.82-0.99)	0.847
TGF-beta	-0.13 (-1.61-1.34)	0.844	-0.27 (-1.52-0.99)	0.651

Supplemental Table S3: Holm-Bonferroni a levels for multiple testing in echocardiographic parameters

**Death or arrhythmia**			**Death or intervention**		
**Parameter**	**p-value**	**α-level according to Holm-Bonferroni method**	**Parameter**	**p-value**	**α-level according to Holm-Bonferroni method**
**LVEF**	0,996	0,005	**GLS**	0,996	0,005
**E/A ratio**	0,533	0,005	**LVESDi**	0,958	0,005
**LVEDDi**	0,302	0,006	**E/E' ratio**	0,841	0,006
**AoV Vmax**	0,170	0,006	**E/A ratio**	0,550	0,006
**AoR (mod/sev)**	0,109	0,007	**LVEDDi**	0,460	0,007
**GLS**	0,096	0,008	**LVEF**	0,064	0,008
**LVESDi**	0,065	0,010	**LAVI**	0,051	0,010
**E/E' ratio**	0,037	0,013	**LV mass inex**	0,010	0,013
**LAVI**	0,006	0,017	**LA enlarged**	0,010	0,017
**LV-mass index**	0,004	0,025	**AoR (mod/sev)**	0,002	0,025
**LA enlarged**	0,002	0,050	**AoV Vmax**	0,001	0,050

Supplemental Table S4: Holm-Bonferroni a levels for multiple testing in blood biomarkers

**Death or arrhythmia**			**Death or intervention**		
**Biomarker**	**p-value**	**α-level according to Holm-Bonferroni method**	**Biomarker**	**p-value**	**α-level according to Holm-Bonferroni method**
**TGF-beta**	0,747	0,008	**CRP**	0,921	0,008
**RDW**	0,381	0,010	**Creatinine**	0,54	0,010
**CRP**	0,259	0,013	**RDW**	0,539	0,013
**Creatinine**	0,156	0,017	**TGF-beta**	0,375	0,017
**TroponinT**	0,065	0,025	**TroponinT**	0,116	0,025
**NT-proBNP**	0,005	0,050	**NT-proBNP**	0,002	0,050

Supplemental Table S5: Sensitivity analysis excluding patients with Turner syndrome or history of aortic coarctation

	Death or arrhythmia		Death or intervention	
	**HR**	**95%-CI**	**HR**	**95%-CI**
**RDW**	0.82	0.44-1.55	1.25	0.73-2.16
**Creatinine**	1.93	0.66-5.70	0.51	0.25-1.03
**C-reactive protein**	1.27	0.68-2.36	1.00	0.65-1.54
**TroponinT**	1.52	0.93-2.48	1.20	0.76-1.86
**NT-proBNP**	2.12	1.31-3.46	2.86	1.70-4.85
**TGF-beta**	0.89	0.41-1.92	0.78	0.36-1.68
**LVEDD**	3.25	1.42-7.39	1.07	0.64-1.79
**LVESD**	1.67	0.81-3.42	0.99	0.63-1.58
**LA volume / m2**	1.06	1.00-1.12	1.03	0.98-1.08
**LA (enlarged)**	7.69	1.80-32.79	2.64	1.03-6.75
**E/A ratio**	1.12	0.51-2.44	1.15	0.68-1.93
**E/E' ratio**	2.56	1.21-5.42	1.05	0.57-1.92
**LVEF**	1.07	0.57-1.99	1.72	1.03-2.83
**GLS**	0.72	0.56-0.93	0.76	0.62-0.92
**LV mass index**	2.83	1.20-6.62	1.88	1.19-2.97
**AoV Vmax**	1.27	0.72-2.27	2.10	1.32-3.32
**AoR (moderate or severe)**	1.90	0.59-6.05	3.16	1.26-7.92
